# Characteristics of Soil Physicochemical Properties and Microbial Community of Mulberry (*Morus alba* L.) and Alfalfa (*Medicago sativa* L.) Intercropping System in Northwest Liaoning

**DOI:** 10.3390/microorganisms11010114

**Published:** 2023-01-01

**Authors:** Muzi Li, Yawei Wei, You Yin, Wenxu Zhu, Xuejiao Bai, Yongbin Zhou

**Affiliations:** 1 Institute of Modern Agricultural Research, Dalian University, Dalian 116622, China; 2 Shenyang Agricultural University, Shenyang 110866, China; 3 Research Station of Liaohe-River Plain Forest Ecosystem, Chinese Forest Ecosystem Research Network (CFERN), Shenyang Agricultural University, Tieling 112000, China; 4 Life Science and Technology College, Dalian University, Dalian 116622, China

**Keywords:** intercropping, *Morus alba* L., *Medicago sativa* L., ecological stoichiometry, soil microorganism

## Abstract

Medicinal plant intercropping is a new intercropping method. However, as a new intercropping model, the influence of intercropping of alfalfa on microorganisms has not been clarified clearly. In this study, the composition and diversity of microbial communities in alfalfa intercropping were studied, and the differences of bacterial and fungal communities and their relationships with environmental factors are discussed. Intercropping significantly decreased soil pH and significantly increased soil total phosphorus (TP) content, but did not increase soil total carbon (TC) and total nitrogen (TN). Intercropping can increase the relative abundance of Actinobacteria and reduce the relative abundance of Proteobacteria in soil. The relative abundance and diversity of bacteria were significantly correlated with soil pH and TP, while the diversity of fungi was mainly correlated with TC, TN and soil ecological stoichiometry. The bacterial phylum was mainly related to pH and TP, while the fungal phylum was related to TC, TN, C: P and N: P. The present study revealed the stoichiometry of soil CNP and microbial community characteristics of mulberry-alfalfa intercropping soil, clarified the relationship between soil stoichiometry and microbial community composition and diversity, and provided a theoretical basis for the systematic management of mulberry-alfalfa intercropping in northwest Liaoning.

## 1. Introduction

Under the background of high quality development of animal husbandry, herbivorous animal husbandry has higher requirements for the yield and quality of herbage [1]. In addition, due to the degradation of natural grassland, China’s pasture production cannot meet the needs of animal husbandry [2]. Alfalfa has strong palatability and contains a lot of protein in its stems and leaves. It is the best feed for laying hens [3], dairy cows and other animals. As a perennial forage, alfalfa can greatly reduce the initial input [4]. Therefore, the demand for high quality alfalfa increases rapidly [5]. Southern China is not suitable for alfalfa planting [6]. However, in the semi-arid area in northern China with the same period of rain and heat, alfalfa can grow well in the saline-alkali environment in northern Liaoning and reduce the nitrogen input in the intercropping system [7]. Intercropping of mulberry-alfalfa is a new method of forest intercropping in recent years [8]. Mulberry is a kind of medicinal plant. Its roots [9], leaves [10] and phellinus [11] can be used as medicine. Its leaves and young stems are also superior tree fodder forages [12]. The protein contained in mulberry leaves is about 15–28%, which is the highest content of the tree fodder forages [13]. The phenolic compounds in mulberry leaves can resist the infection of some pathogens and insects [14], and the abundant bioactive substances can enhance the immunity of the body [15].

It is well known that intercropping is achieved through different plant configurations to improve productivity or ecological benefits [16,17], and the internal mechanism of this facilitation is driven by soil microorganisms [18]. For aboveground vegetation, soil microorganisms (bacteria and fungi) use their own characteristics to shape plant diversity [19], promote plant growth [20], improve plant productivity, and participate in plant decomposition [21]. For underground ecological processes, soil microorganisms can affect plant allelopathy [22], regulate organic matter accumulation [23], and promote the absorption and transformation of soil nutrients [24,25].

At present, studies on the intercropping of mulberry and alfalfa mainly focus on the effects of the intercropping system and fertilization on soil physicochemical properties and microbial community structure, but there are few studies on the relationship between environmental factors and soil microorganisms [26]. The relevant research of Zhang et al. is different from the test area of this study, resulting in a large difference in microbial composition and structure [8,27]. Previous studies mainly focused on soil available nutrients, while this study was elaborated from soil stoichiometry and microbial community. Moreover, the number of OTUs detected in this study is much higher than that in previous studies, and the data are more convincing. Soil is the living place of plants and microorganisms, and the change of soil environment has a high correlation with microorganisms [7]. In order to clarify the relationship between environmental factors and microbial communities under intercropping conditions, in this study, we used high-throughput sequencing technology to determine the soil microbial community composition of mulberry-alfalfa intercropping. Combined with the analysis of the soil’s physical and chemical properties and ecological stoichiometry characteristics, the response model of the soil’s physical and chemical properties, ecological stoichiometry and soil microorganisms to mulberry-alfalfa intercropping was determined, and the correlation between soil C, N and P elements and their stoichiometry characteristics and microorganisms in this system was explored. It provides theoretical support for the establishment of a management system for the mulberry-alfalfa intercropping system.

## 2. Materials and Methods

### 2.1. Test Varieties and Planting Patterns

The pilot area was located in Zhangwu County, northwest Liaoning Province, China (42°07′–42°51′ N, 121°53′–122°58′ E). The experiment was conducted in the Breeding Center of Zhangwu County Aerospace Park. Mulberry is the variety “Shensang No. 1” cultivated by the Forestry College of Shenyang Agricultural University, and alfalfa is the “Queen” of alfalfa cultivated in Paozi Town of Fumeng County. The soil used in the experiment was taken from the same block, and its physicochemical properties were as follows: organic carbon content was 4.74 g kg^−1^, TN content was 0.44 g kg^−1^, and TP content was 0.50 g kg^−1^. Three plant configurations were established in this experiment: mulberry monoculture (MM), alfalfa monoculture (MA) and mulberry-alfalfa intercropping (IMA). The upper inner diameter of the pot was 25 cm, the bottom inner diameter was 17 cm, and the height was 30 cm. Potted plants were placed on the same plot at random. “MM” has two mulberries per pot, “MA” has four alfalfas per pot, “IMA” has one mulberry and two alfalfas per pot. Four replicates were set in this experiment. The annual mulberry seedlings and alfalfa seedlings were planted on 8 April 2021. In the experiment, the system was not fertilized, only weeds were removed.

### 2.2. Collection and Chemical Property Analysis of Soil Samples

The plant was removed from the basin, the soil outside its root was shaken off, and the soil attached to the root was taken for subsequent measurement. The experiment consisted of three treatments and 12 samples. The soil was placed in a bag and stored in an ice box. After returning to the laboratory, the soil debris was removed and screened with a 100 mesh (diameter 0.150 mm) screen. The soil samples for the determination of physical and chemical properties were air-dried and stored at 4 °C. The soil samples for the determination of soil microorganisms were packed into a centrifugal tube and stored in a refrigerator at −180 °C.

Instruments required for soil physical and chemical properties determination are as follows: soil pH, Leici PHS-3C pH meter; soil total carbon (TC) and nitrogen (TN), elemental analyzer (Elementar Vario EL III, Hanau, Germany); Soil total phosphorus (TP), AA3 flow analyzer (AA3, Hamburg, Germany).

### 2.3. DNA Extraction and Sequencing of Soil Microorganisms

The total genomic DNA of soil microorganisms was extracted by OMEGA Mag-bind Soil DNA Kit (Omega M5636–02) (Omega Bio-Tek, Norcross, GA, USA). After qualitative detection by agarose gel electrophoresis, the concentration was determined by a nucleic acid quantifier NanoDrop ND-1000 (Thermo Fisher Scientific, Waltham, MA, USA). Using the extracted total DNA as a template, the V3–V4 region of the bacterial 16S rRNA gene was amplified with primers 338F (5′–ACTCCTACGGGAGGCAGCAG–3′) and 806R (5′–GGACTACHVGGGTWTCTAAT–3′). The fungal ITS region was amplified with primers ITS1F (5′–CTTGGTCATTTAGAGGAAGTAA–3′) and ITS2R (5′–GCTGCGTTCTTCATCGATGC–3′). PCR amplification and sequencing was performed by Shanghai Paisonol Biotechnology Co., Ltd. TruSeq Nano DNA LT library Prep Kit (Illumina) (Shanghai, China).

### 2.4. Statistical Analysis

IBM SPSS Statistics 22.0 (Chicago, IL, USA) was used for statistical analysis of experimental data. Univariate analysis of variance (ANOVA) was used to analyze the significance of differences. Microbiome bioinformatics were performed with QIIME2 2019.4 [28] with slight modification according to the official tutorials (https://docs.qiime2.org/2019.4/tutorials/ (accessed on 1 November 2019)). The steps of priming removal, mass filtering, denoise, splicing and dechimerism were implemented using DADA2 methods [29]. Each sequence of weight removal generated after quality control is called ASVs (corresponding to OTU representing sequence). ASVs used the classify-sklearn naïve Bayes taxonomy classifier in feature-classifier plugin [30] against the SILVA Release 132 (Bacteria)/UNITE Release 8.0 (Fungi) Database [31]. The self-written perl script was used to calculate the number of taxon contained in different samples at each classification level, and the feature table after singleton was removed, counted and plotted with “qiime taxa barplot”. The Venn diagrams were generated using the “VennDiagram” package in R (R v.3.4.4) (New Zealand). The alpha diversity and beta diversity were analyzed using QIIME (Version 1.7.0) software package. The data normalization process was used in the alpha diversity analysis. The “p-min-depth” was set to 10 and the “p-max-depth “was set to 95% of the minimum sequencing depth in all samples. The alpha diversity of species was characterized by Shannon, Simpson, Pielou’s evenness and Observed_species. The MetaCyc database was used to predict the metabolic pathway of 16S rRNA and ITS gene sequences, and Bray–Curtis distance combined with principal coordinates analysis was used to visualize the functional differences of samples. Bray–Curtis distance was calculated using the “qiime diversity core-metrics” command. CANOCO 5.0 software (http://www.canoco5.com/ (accessed on 5 December 2022)) was used to analyze redundancy among environmental factors, microbial abundance changes and environmental factors.

## 3. Results

### 3.1. Effects of Mulberry-Alfalfa Intercropping on Soil CNP Stoichiometry

Soil pH of the intercropping was significantly lower than that of the monoculture. The contents of TC and TN in MA soil, as well as C: P and N: P in soil were significantly higher than those in MM and IMA. The soil TP content of IMA was the highest, and the TP content of MM was significantly different from that of MA, but there was no significant difference. The soil C: N of IMA and MA was significantly higher than that of MM (Table 1).

### 3.2. Effects of Mulberry-Alfalfa Intercropping on Soil Microflora Composition and Structure

**Figure 1:**
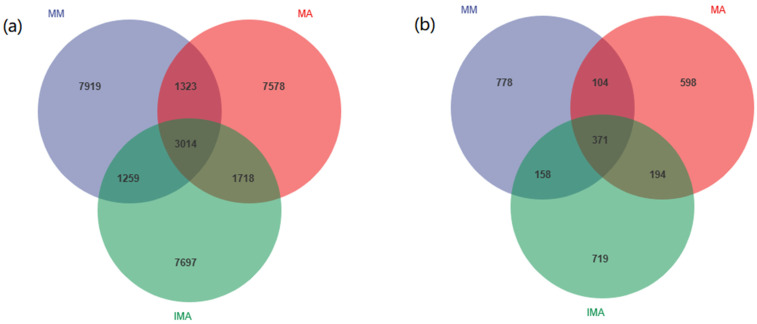
OTUs Venn diagrams. (**a**): OTUs detected in bacteria; (**b**): OTUs detected in fungi. MM: *Morus alba*; MA: *Medicago sativa*; IMA: *Morus alba*–*Medicago sativa*.

With Paired-end sequencing, a total of 904,537 effective tags were obtained from 12 soil samples. A total of 33,430 OTUs were detected in all samples, including 30,508 OTUs for bacteria and 2922 OTUs for fungi. Bacterial OTU analysis showed that 3014 OTUs were common to all samples, 1323 OTUs were shared by mulberry monocropping (MM) and alfalfa monocropping (MA), 1259 OTUs were shared by mulberry monocropping and mulberry-alfalfa intercropping, 1718 OTUs were shared by alfalfa monocropping and mulberry-alfalfa intercropping (Figure 1a). Fungal OTU analysis showed that 371 OTUs were common to all samples, 104 OTUs were found in mulberry monoculture (MM) and alfalfa monoculture (MA), 158 OTUs were found in mulberry monoculture and mulberry-alfalfa intercropping, and 194 OTUs were found in alfalfa monoculture and mulberry-alfalfa intercropping (Figure 1b).

**Figure 2:**
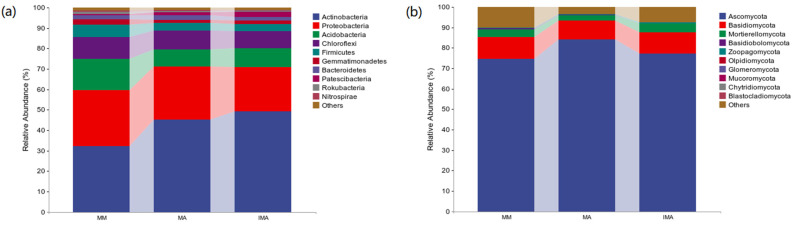
Relative abundance of species composition at the soil microbial phylum level in different treatments. (**a**): Relative abundance of bacterial phylum-level species composition; (**b**): Relative abundance of fungal phylum-level species composition. MM: *Morus alba*; MA: *Medicago sativa*; IMA: *Morus alba*–*Medicago sativa*.

In the bacterial community, there are four dominant phylas, namely Actinobacteria, Proteobacteria, Acidobacteria and Chloroflexi (Figure 2a). Actinobacteria maintained the highest relative abundance in the three groups. Its relative abundance in IMA and MA was significantly higher than that in MM (*p* < 0.05). The relative abundance of Proteobacteria in IMA was significantly lower than that in the other two treatments (*p* < 0.05). Chloroflexi and Acidobacteria in MM had the highest relative abundance (15% and 11%, respectively), which was significantly higher than that of IMA and MA (*p* < 0.05) (Table 2). In the fungal community, the relative abundance of ascomycota in each treatment was more than 70%, which was in an absolutely dominant position (Figure 2b). The relative abundance of ascomycetes of MA was 84%, which was significantly higher than MM (*p* < 0.05), but it had no significant difference with IMA (*p* > 0.05) (Table 2).

**Figure 3:**
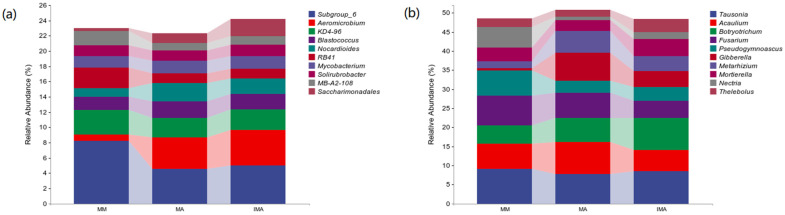
Relative abundance of species composition at the soil microbial genus level in different treatments. (**a**): Relative abundance of bacterial genus-level species composition; (**b**): Relative abundance of fungal genus-level species composition. MM: *Morus alba*; MA: *Medicago sativa*; IMA: *Morus alba*–*Medicago sativa*.

The community structure of bacteria (fungi) under different treatments was significantly different at genus level. Subgroup_6 and *Aeromicrobium* prevailed in the community (relative abundance > 5%), but their abundance distributions were different in different treatments (Figure 3a). In MM, the relative abundance of *Subgroup_6* was significantly higher than that of IMA and MA (*p* < 0.05), while that of *Aeromicrobium* was significantly lower than that of other treatments (*p* < 0.05). There was no significant difference between IMA and MA (Table 3). There are six dominant genera of fungi (Figure 3b). Tausonia, *Acaulium* and *Fusarium* showed no significant differences among the three treatments. The relative abundance of *Botryotrichum* in IMA was significantly higher than that of MM, that of *Pseudogymnoascus* was significantly higher than that of IMA, and that of *Gibberella* was the highest in MA but not significantly different from that of IMA (Table 3).

**Figure 4:**
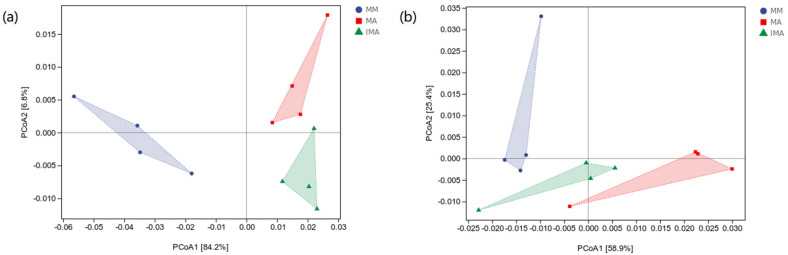
Principal coordinates analysis of potential functions of soil microorganisms under different treatments. Principal coordinates analysis plot based on the Bray–Curtis distance. (**a**): soil bacterial communities; (**b**): soil fungal communities. MM: *Morus alba*; MA: *Medicago sativa*; IMA: *Morus alba*–*Medicago sativa*.

There was no significant difference in the normalized abundance of functional units of bacteria and fungi. Therefore, we used the sample differential distance matrix combined with principal coordinate analysis (PcoA) to expand the sample functional differences in a low dimension. The first axis of bacterial functional unit PcoA explained 84.2%, and the second axis only explained 6.8%. IMA and MA are located on the same side of the first axis and are far away from MM (Figure 4a). The first axis of fungal functional unit PcoA explained 58.9%, and the second axis explained 25.4%. IMA and MA are located on the same side of the second axis and are far away from MM. The three groups were treated at similar distances (Figure 4b). In general, MA and IMA have higher similarities in bacterial and fungal functions.

### 3.3. The Impact of Mulberry-Alfalfa Intercropping on Diversity of Soil Microbial Populations

**Figure 5:**
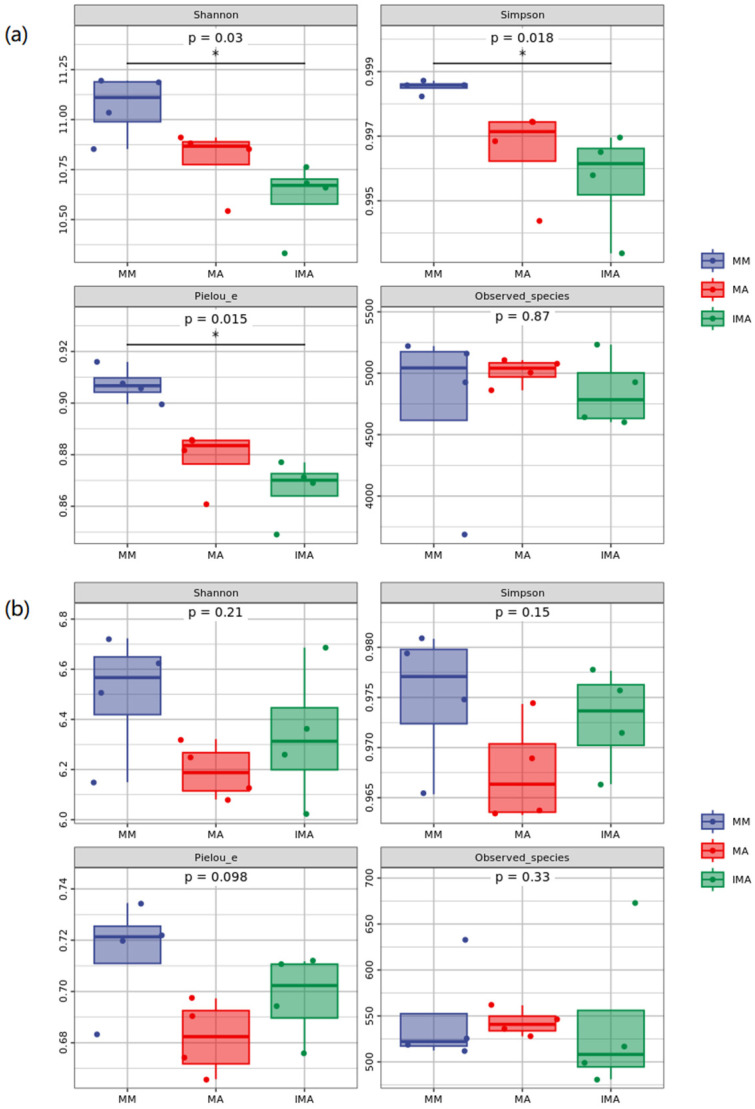
Alpha diversity of soil microorganisms under different treatments. (**a**): Analysis of alpha diversity of soil bacterial community; (**b**): Analysis of alpha diversity of soil fungal community. * Correlation is significant at the 0.05 level. MM: *Morus alba*; MA: *Medicago sativa*; IMA: *Morus alba*–*Medicago sativa*.

In Figure 5a, Shannon, Simpson and Pielou’s evenness indexes of soil bacteria showed significant differences. The above index of three groups of intermediate cropping was significantly lower than that of mulberry and alfalfa monocropping. As shown in Figure 5b, except for Observed species, the diversity indices of MA in soil fungi are all low, MM is high, and LB is in the middle.

### 3.4. Correlation Analysis between Soil Physical and Chemical Properties and Soil Microorganisms

As shown in Table 4, there is a closer correlation between fungal diversity and soil physicochemical properties. In bacterial diversity, Simpson and Pielou_e’s indices were significantly correlated with pH and TP, while fungal diversity was not significantly correlated with these two physicochemical properties. Fungal diversity was negatively correlated with most physical and chemical properties.

**Figure 6:**
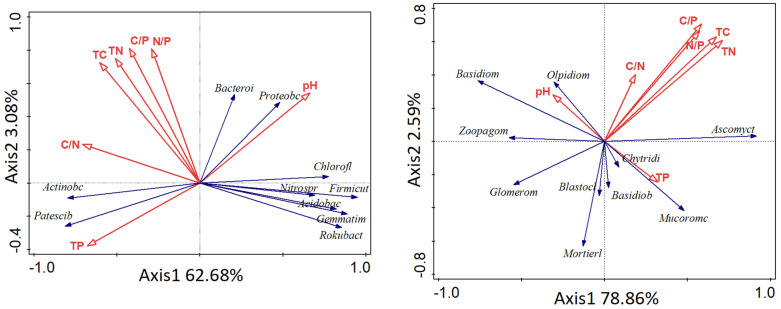
Correlation between soil physical and chemical properties and soil microorganisms.

Redundancy analysis (RDA) was used to further analyze the effects of soil physical and chemical properties on soil microbial community structure. The first two axes of bacterial RDA explained 62.68% and 3.08% of the total variance, respectively, for a total of 65.76%. Soil pH (*p* = 0.042) and TP (*p* = 0.044) were the most important factors affecting the soil bacterial community. Soil pH was negatively correlated with Actinobacteria and positively correlated with Chloroflexi, Proteobacteria and Acidobacteria. The correlation between TP and the above phylum was the opposite (Figure 6a). The first two axes of fungal RDA explained 78.86% and 2.59% of the total variance, respectively, accounting for 81.44%. Soil TN (*p* = 0.020) and TC (*p* = 0.040) were the most important factors affecting the soil fungal community, and they were positively correlated with Ascomycota (Figure 6b).

## 4. Discussion

*Medicago sativa* is one of the common herbages in arid and semi-arid areas, and as a leguminous crop, alfalfa has a certain nitrogen fixation effect [32]. Many early studies have shown that intercropping forage legumes with other species can increase the yield of other plants [33]. In addition, intercropping with leguminous crops can improve the system’s fixation of atmospheric nitrogen. In the long run, nitrogen will spread to neighboring plants and increase the concentration and accumulation of nitrogen in the rhizosphere [34,35,36]. This biological nitrogen fixation method effectively reduces the need for nitrogen fertilizer and alleviates environmental pressure [37,38,39]. In our experiment, TC and TN contents of the alfalfa monoculture were significantly higher than those of the mulberry monoculture and intercropping, which was consistent with the results of the above related studies. Although the TN content in intercropping was not significantly different from that in the mono-cropping of mulberry, it still increased slightly. This increase in nitrogen may be due to the fact that most nitrogen transfer is through direct contact with roots [40], In this experiment, pot cultivation increased the root contact area of mulberry and alfalfa, and the transfer amount between them increased. Soil phosphorus is an important limiting factor for legume yield [41]. Oelmann reported that intercropping was more efficient than monocropping in phosphorus utilization [42]. In this study, intercropping soil can obtain more soil P to ensure the P supply of the intercropping system. The pH of intercropping soil decreased, which was consistent with the results of Zhang *et al.* [7].

Surface litter, soil microorganisms and soil nutrients all affect C, N and P ecological stoichiometry. The C: N of mulberry monocropping was low, which was due to the relatively small amount of plant litter in the soil under this treatment, resulting in the low TC content in the soil. The migration ability of the soil P element between plants and soil can be determined by soil C: P [43]. In this experiment, the C: P of intercropping was significantly lower than that of alfalfa monocropping, indicating that the P availability of intercropping soil was higher than that of alfalfa monocropping, and the higher P availability was enough to improve crop productivity [44]. Soil N: P is often used to determine nutrient limiting thresholds and nitrogen saturation [45]. N: P in the three groups was lower than the national level (9.3) [46], and the soil was still limited by nitrogen.

At phylum level, the structure of bacterial and fungal communities was similar in all treatments, but the relative abundance was different. Among the dominant bacterial phyla, compared with mulberry monoculture, intercropping significantly increased the relative abundance of Actinobacteria, while significantly reducing the relative abundance of Proteobacteria, Acidobacteria and Chloroflexi. The soil of the experimental area is weakly alkaline, which is the most suitable environment for the growth of Actinobacteria [47]. The increase in the relative abundance of Actinobacteria promotes the mineralization of TC [48,49]. However, Acidobacteria is acidophilus and has low competitiveness in this environment [50]. The relative abundance of Acidobacteria in mulberry monoculture soil is high and the tolerance of mulberry to environmental pressure is strong [51]. The tolerance of alfalfa is weak, and intercropping can improve its tolerance to some extent. Ascomycota dominated the fungal community, which was related to the carbon and nitrogen cycle and could degrade plants and improve soil stability [52]. The abundance of Ascomycota in intercropped soil was lower than that of alfalfa monoculture, but the difference was not significant.

The relative abundance of bacteria and fungi decreased by one order of magnitude compared with the phylum level, and there were great differences in each treatment. *Subgroup_6* belongs to Proteobacteria, which is a kind of oligotrophic bacteria which is negatively correlated with soil nutrients [53]. The relative abundance of alfalfa in mono-cropping and intercropping soils was low, indicating that intercropping could maintain a higher level of soil nutrients. *Aeromicrobium* belongs to the phylum actinomycetes and has disease-suppressing properties [54]. It accumulates in intercropping soils. The intercropping system contained a high abundance of *Botryotrichum*, which was mostly saprophytic fungi, which increased litter decomposition in the intercropping system.

Through the principal coordinate analysis of microbial functional units, the functions of bacteria and fungi in each treatment were significantly different. The bacterial functions of intercropping and alfalfa monocropping were more similar, indicating that alfalfa rhizosphere bacteria were dominant in the intercropping system. There was no significant distance difference between fungi in the first axis, while intercropping and alfalfa monoculture were closer to each other in the second wheelbase. However, due to the small amount of interpretation in the second axis, they were not sufficiently convincing.

The bacterial diversity and evenness of the three groups were significantly different, and the alpha diversity of intercropping was lower. pH was positively correlated with bacterial diversity and evenness, while diversity was negatively correlated with TP. There was no significant difference in fungal diversity, which may be due to the soil with the same origin in the three groups [55]. The alpha diversity of intercropping of mulberry was still the highest, and the alpha diversity of intercropping was higher than that of alfalfa [56], indicating that the increase in plant diversity could improve the diversity of forage fungi [57]. Fungal alpha diversity was mainly related to TC, TN and soil ecological stoichiometry [58]. This result is consistent with the results of Sanaei et al., bacterial diversity is related to carbon sources, and fungal diversity is related to soil stoichiometry [59]. RDA results showed that the bacterial community was significantly correlated with soil nutrients and stoichiometry, and pH was positively correlated with more bacterial phyla [60]. TP is the most important factor affecting Actinobacteria. This is not completely consistent with Sanyika’s results on soil determinants of actinomycetes community structure [61]. TC, TN, C: P and N: P were the main influencing factors of the fungal community, and were significantly positively correlated with the dominant phylum Ascomycota, while negatively correlated with most phylum fungi. The effect of pH and TP on the fungal community was smaller than that on the bacterial community. However, the interpretation quantity of the second axis of RDA plots in both groups is low.

## 5. Conclusions

This study mainly studied the changes of soil physicochemical properties and microbial community structure of mulberry-alfalfa intercropping in northwest Liaoning. The soil type in this area is relatively barren aeolian sandy soil. The results showed that intercropping could improve the soil environment in this area by changing soil pH and stoichiometry, and increasing the relative abundance of beneficial microorganisms. This study provides theoretical guidance for the restoration of alkaline aeolian sandy soil in northwest Liaoning. Further studies are needed to determine the microbial driving mechanism of the intercropping system under long-term tillage conditions.

## Figures and Tables

**Table 1:** C, N, P content and ecological stoichiometry in rhizosphere soils.

Soil Chemistry	MM	MA	IMA
pH Value	7.91 ± 0.03 a	7.86 ± 0.01 a	7.74 ± 0.05 b
TC (g kg^−1^)	4.54 ± 0.18 c	8.97 ± 0.39 a	5.60 ± 0.26 b
TN (g kg^−1)^	0.47 ± 0.01 b	0.82 ± 0.06 a	0.52 ± 0.01 b
TP (g kg^−1^)	0.44 ± 0.01 b	0.47 ± 0.03 ab	0.51 ± 0.01 a
C: N	9.70 ± 0.19 b	11.08 ± 0.45 a	10.75 ± 0.27 a
C: P	10.42 ± 0.62 b	19.12 ± 1.40 a	11.11 ± 0.76 b
N: P	1.07 ± 0.05 b	1.75 ± 0.19 a	1.03 ± 0.05 b

The data in the table are mean ± standard error; the lowercase letters denote significant differences between the treatments determined by Duncan’s tests (*p* < 0.05). MM: *Morus alba*; MA: *Medicago sativa*; IMA: *Morus alba*–*Medicago sativa*.

**Table 2:** Effects of intercropping on the relative abundance of phylum level dominant bacteria and fungi.

Phylum		MM	MA	IMA
Bacteria	Actinobacteria	0.32 ± 0.03 b	0.45 ± 0.01 a	0.49 ± 0.01 a
	Proteobacteria	0.27 ± 0.02 a	0.26 ± 0.01 a	0.22 ± 0.01 b
	Acidobacteria	0.15 ± 0.01 a	0.08 ± 0.01 b	0.09 ± 0.01 b
	Chloroflexi	0.11 ± 0.01 a	0.09 ± 0.01 b	0.08 ± 0.01 b
Fungi	Ascomycota	0.75 ± 0.02 b	0.84 ± 0.019 a	0.77 ± 0.03 ab

The data in the table are mean ± standard error; The lowercase letters denote significant differences between the treatments determined by Duncan’s tests (*p* < 0.05). MM: *Morus alba*; MA: *Medicago sativa*; IMA: *Morus alba*–*Medicago sativa*.

**Table 3:** Effects of intercropping on relative abundance of genus level dominant bacteria and fungi.

Genus		MM	MA	IMA
Bacteria	*Subgroup_6*	0.08 ± 0.01 a	0.05 ± 0.01 b	0.05 ± 0.01 b
	*Aeromicrobium*	0.01 ± 0.01 b	0.04 ± 0.01 a	0.05 ± 0.01 a
Fungi	*Tausonia*	0.09 ± 0.01 a	0.08 ± 0.01 a	0.08 ± 0.01 a
	*Acaulium*	0.07 ± 0.01 a	0.08 ± 0.01 a	0.06 ± 0.01 a
	*Botryotrichum*	0.05 ± 0.01 b	0.06 ± 0.01 ab	0.08 ± 0.01 a
	*Fusarium*	0.08 ± 0.01 a	0.07 ± 0.02 a	0.04 ± 0.01 a
	*Pseudogymnoascus*	0.07 ± 0.01 a	0.03 ± 0.01 ab	0.04 ± 0.01 b
	*Gibberella*	0.01 ± 0.01 b	0.07 ± 0.02 a	0.04 ± 0.01 ab

The data in the table are mean ± standard error; The lowercase letters denote significant differences between the treatments determined by Duncan’s tests (*p* < 0.05). MM: *Morus alba*; MA: *Medicago sativa*; IMA: *Morus alba*–*Medicago sativa*.

**Table 4:** Relationship between soil physicochemical properties and stoichiometry and alpha diversity of soil microbial community.

Alpha Diversity		pH	TC	TN	TP	C/N	C/P	N/P
Bacteria	Shannon	0.113	0.210	0.207	0.119	0.103	0.178	0.159
	Simpson	0.604 *	−0.416	−0.332	−0.641 *	−0.555	−0.234	−0.120
	Pielou_e	0.625 *	−0.304	−0.224	−0.54 8	−0.490	−0.147	−0.046
	Observed_species	0.528	−0.374	−0.301	−0.563	−0.480	−0.203	−0.104
Fungi	Shannon	−0.124	−0.105	−0.024	−0.042	−0.400	−0.100	−0.021
	Simpson	−0.018	−0.754 **	−0.693 *	−0.089	−0.611 *	−0.710 **	−0.613 *
	Pielou_e	−0.058	−0.649 *	−0.567	−0.095	−0.660 *	−0.610 *	−0.499
	Observed_species	−0.177	−0.674 *	−0.611 *	−0.001	−0.574	−0.655 *	−0.563

* Correlation is significant at the 0.05 level; ** Correlation is significant at the 0.01 level.

## Data Availability

The data presented in this study are available on request from the corresponding author. The data are not publicly available due to the policy of the institute.

## References

[1] Hou L., Bai W., Zhang Q., Liu Y., Sun H., Luo Y., Song S., Zhang W.-H. (2022). A new model of two-sown regime for oat forage production in an alpine region of northern China. Environ. Sci. Pollut. Res..

[2] Zhang W., Hou L., Yang J., Song S., Mao X., Zhang Q., Bai W., Pan Q., Zhou Q. (2018). Establishment and management of alfalfa pasture in cold regions of China. Chin. Sci. Bull..

[3] Wüstholz J., Carrasco S., Berger U., Sundrum A., Bellof G. (2017). Silage of young harvested alfalfa (*Medicago sativa*) as home-grown protein feed in the organic feeding of laying hens. Org. Agric..

[4] Rock K.P., Thelemann R.T., Jung H.-J.G., Tschirner U.W., Sheaffer C.C., Johnson G.A. (2009). Variation due to Growth Environment in Alfalfa Yield, Cellulosic Ethanol Traits, and Paper Pulp Characteristics. BioEnergy Res..

[5] Wang B., Zhao F., Zhang B., Liu J. (2016). An insufficient glucose supply causes reduced lactose synthesis in lactating dairy cows fed rice straw instead of alfalfa hay. J. Anim. Sci..

[6] Dai Q., Hou Z., Gao S., Li Z., Wei Z., Wu D. (2019). Substitution of fresh forage ramie for alfalfa hay in diets affects production performance, milk composition, and serum parameters of dairy cows. Trop. Anim. Health Prod..

[7] Zhang X., Teng Z., Zhang H., Cai D., Zhang J., Meng F., Sun G. (2021). Nitrogen application and intercropping change microbial community diversity and physicochemical characteristics in mulberry and alfalfa rhizosphere soil. J. For. Res..

[8] Zhang M., Wang N., Hu Y., Sun G. (2018). Changes in soil physicochemical properties and soil bacterial community in mulberry (*Morus alba* L.)/alfalfa (*Medicago sativa* L.) intercropping system. Microbiol. Open.

[9] Inyai C., Yusakul G., Komaikul J., Kitisripanya T., Likhitwitayawuid K., Sritularak B., Putalun W. (2021). Improvement of stilbene production by mulberry Morus alba root culture via precursor feeding and co-elicitation. Bioprocess Biosyst. Eng..

[10] Zhang Z., Zhang Y., Zhang S., Wang L., Liang X., Wang X., Wu H., Zou H., Zhang C., Wang M. (2022). Foliar Spraying of 6-Benzylaminopurine Promotes Growth and Flavonoid Accumulation in Mulberry (*Morus alba* L.). J. Plant Growth Regul..

[11] Ma Y., Gao W., Zhang F., Zhu X., Kong W., Niu S., Gao K., Yang H. (2022). Community composition and trophic mode diversity of fungi associated with fruiting body of medicinal *Sanghuangporus vaninii*. BMC Microbiol..

[12] Simbaya J., Chibinga O., Salem A.Z.M. (2020). Nutritional evaluation of selected fodder trees: Mulberry (*Molus alba* Lam.), Leucaena (*Leucaena luecocephala* Lam de Wit.) and Moringa (*Moringa oleifera* Lam.) as dry season protein supplements for grazing animals. Agrofor. Syst..

[13] Wang B., Luo H. (2021). Effects of mulberry leaf silage on antioxidant and immunomodulatory activity and rumen bacterial community of lambs. BMC Microbiol..

[14] Krajnc A.U., Ugulin T., Paušič A., Rabensteiner J., Bukovac V., Petkovšek M.M., Janžekovič F., Bakonyi T., Berčič R.L., Felicijan M. (2019). Morphometric and biochemical screening of old mulberry trees (*Morus alba* L.) in the former sericulture region of Slovenia. Acta Soc. Bot. Pol..

[15] He X., Fang J., Ruan Y., Wang X., Sun Y., Wu N., Zhao Z., Chang Y., Ning N., Guo H. (2018). Structures, bioactivities and future prospective of polysaccharides from *Morus alba* (white mulberry): A review. Food Chem..

[16] Dai C.-C., Chen Y., Wang X.-X., Li P.-D. (2013). Effects of intercropping of peanut with the medicinal plant *Atractylodes lancea* on soil microecology and peanut yield in subtropical China. Agrofor. Syst..

[17] Li H., Luo L., Tang B., Guo H., Cao Z., Zeng Q., Chen S., Chen Z. (2022). Dynamic changes of rhizosphere soil bacterial community and nutrients in cadmium polluted soils with soybean-corn intercropping. BMC Microbiol..

[18] Fierer N. (2017). Embracing the unknown: Disentangling the complexities of the soil microbiome. Nat. Rev. Microbiol..

[19] Yang X., Long Y., Sarkar B., Li Y., Lü G., Ali A., Yang J., Cao Y.-E. (2021). Influence of soil microorganisms and physicochemical properties on plant diversity in an arid desert of Western China. J. For. Res..

[20] Solanki M.K., Wang F.-Y., Wang Z., Li C.-N., Lan T.-J., Singh R.K., Singh P., Yang L.-T., Li Y.-R. (2019). Rhizospheric and endospheric diazotrophs mediated soil fertility intensification in sugarcane-legume intercropping systems. J. Soils Sediments.

[21] van der Heijden M.G.A., Wagg C. (2013). Soil microbial diversity and agro-ecosystem functioning. Plant Soil.

[22] Cipollini D., Rigsby C.M., Barto E.K. (2012). Microbes as Targets and Mediators of Allelopathy in Plants. J. Chem. Ecol..

[23] Julia K., Karina E.C., Erik K., Björn L.D. (2017). Below-ground organic matter accumulation along a boreal forest fertility gradient relates to guild interaction within fungal communities. Ecol. Let..

[24] Chen Y., Bonkowski M., Shen Y., Griffiths B., Jiang Y., Wang X., Sun B. (2020). Root ethylene mediates rhizosphere microbial community reconstruction when chemically detecting cyanide produced by neighbouring plants. Microbiome.

[25] Urbanová M., Šnajdr J., Baldrian P. (2015). Composition of fungal and bacterial communities in forest litter and soil is largely determined by dominant trees. Soil Biol. Biochem..

[26] Zaeem M., Nadeem M., Pham T.H., Ashiq W., Ali W., Gilani S.S.M., Elavarthi S., Kavanagh V., Cheema M., Galagedara L. (2019). The potential of corn-soybean intercropping to improve the soil health status and biomass production in cool climate boreal ecosystems. Sci. Rep..

[27] Zhang M., Wang N., Zhang J., Hu Y., Cai D., Guo J., Wu D., Sun G. (2019). Soil Physicochemical Properties and the Rhizosphere Soil Fungal Community in a Mulberry (*Morus alba* L.)/Alfalfa (*Medicago sativa* L.) Intercropping System. Forests.

[28] Bolyen E., Rideout J.R., Dillon M.R., Bokulich N.A., Abnet C., Al-Ghalith G.A., Caporaso J.G. (2018). QIIME 2: Reproducible, interactive, scalable, and extensible microbiome data science. PeerJ Prepr..

[29] Callahan B.J., Mcmurdie P.J., Rosen M.J., Han A.W., Johnson A.J.A., Holmes S.P. (2016). DADA_2_: High-resolution sample inference from Illumina amplicon data. Nat. Methods.

[30] Bokulich N.A., Kaehler B.D., Rideout J.R., Dillon M., Bolyen E., Knight R., Huttley G.A., Caporaso J.G. (2018). Optimizing taxonomic classification of marker-gene amplicon sequences with QIIME 2’s q2-feature-classifier plugin. Microbiome.

[31] Kõljalg U., Nilsson R.H., Abarenkov K., Tedersoo L., Taylor A.F.S., Bahram M., Bates S.T., Bruns T.D., Bengtsson-Palme J., Callaghan T.M. (2013). Towards a unified paradigm for sequence-based identification of fungi. Mol. Ecol..

[32] Echeverria A., Gonzalez E.M. (2021). Root system of *Medicago sativa* and *Medicago truncatula*: Drought effects on carbon metabolism. Plant Soil.

[33] Tautges N.E., Jungers J.M., DeHaan L.R., Wyse D.L., Sheaffer C.C. (2018). Maintaining grain yields of the perennial cereal intermediate wheatgrass in monoculture v. bi-culture with alfalfa in the Upper Midwestern USA. J. Agric. Sci..

[34] Crews T.E., Blesh J., Culman S.W., Hayes R.C., Jensen E.S., Mack M.C., Peoples M.B., Schipanski M.E. (2016). Going where no grains have gone before: From early to mid-succession. Agric. Ecosyst. Environ..

[35] Thilakarathna M.S., McElroy M.S., Chapagain T., Papadopoulos Y.A., Raizada M.N. (2016). Belowground nitrogen transfer from legumes to non-legumes under managed herbaceous cropping systems. A review. Agron. Sustain. Dev..

[36] Dimitrova M.L.M., Ana B., Li S., Jensen E.S. (2022). Agronomic performance, nitrogen acquisition and water-use efficiency of the perennial grain crop *Thinopyrum intermedium* in a monoculture and intercropped with alfalfa in Scandinavia. Agron. Sustain. Dev..

[37] Jensen E.S., Carlsson G., Hauggaard-Nielsen H. (2020). Intercropping of grain legumes and cereals improves the use of soil N resources and reduces the requirement for synthetic fertilizer N: A global-scale analysis. Agron. Sustain. Dev..

[38] Carof M., Godinot O., Ridier A. (2019). Diversity of protein-crop management in western France. Agron. Sustain. Dev..

[39] Jensen E.S., Peoples M.B., Boddey R., Gresshoff P.M., Hauggaard-Nielsen H., Alves B.J., Morrison M.J. (2012). Legumes for mitigation of climate change and the provision of feedstock for biofuels and biorefineries. A review. Agron. Sustain. Dev..

[40] Anke H., Franziska N., Thorsten H., Christian B., Jürgen H., Jens D., Georg J.R., Florian W. (2021). Evidence of considerable C and N transfer from peas to cereals via direct root contact but not via mycorrhiza. Sci. Rep..

[41] Latati M., Aouiche A., Rebou Y.N., Laouar M. (2019). Modeling the functional role of the microorganisms in the daily exchanges of carbon and nitrogen in intercropping system under Mediterranean conditions. Agron. Res..

[42] Yvonne O., Markus L., Sophia L., Christiane R., Felipe A., Fabian A., Nina B., Doreen B., Steffen B., Runa B.S. (2021). Above and belowground biodiversity jointly tighten the P cycle in agricultural grasslands. Nat. Commun..

[43] Yadav A., Bhatia A., Yadav S., Kumar V., Singh B. (2019). The effects of elevated CO_2_ and elevated O_3_ exposure on plant growth, yield and quality of grains of two wheat cultivars grown in north India. Heliyon.

[44] Mo F., Han J., Wen X., Wang X., Li P., Vinay N., Jia Z., Xiong Y., Liao Y. (2020). Quantifying regional effects of plastic mulch on soil nitrogen pools, cycles, and fluxes in rain-fed agroecosystems of the Loess Plateau. Land Degrad. Dev..

[45] Lu J., Tian H., Zhang H., Xiong J., Yang H., Liu Y. (2021). Shoot-soil ecological stoichiometry of alfalfa under nitrogen and phosphorus fertilization in the Loess Plateau. Sci. Rep..

[46] Tian H., Chen G., Zhang C., Melillo J.M., Hall C.A.S. (2010). Pattern and variation of C:N:P ratios in China&rsquo;s soils: A synthesis of observational data. Biogeochemistry.

[47] Gohain A., Manpoong C., Saikia R., De Mandal S., Mandal D.S., Bhatt P. (2020). Actinobacteria: Diversity and biotechnological applications. Recent Advancements in Microbial Diversity.

[48] Fu Y., Luo Y., Auwal M., Singh B.P., Van Zwieten L., Xu J. (2022). Biochar accelerates soil organic carbon mineralization via rhizodeposit-activated Actinobacteria. Biol. Fertil. Soils.

[49] Saidi S., Hafsa C.S., Ali C.B., Allaoua S., Manal E., Lenka L., Faizah N.A., Lassaad B. (2021). Improvement of *Medicago sativa* crops productivity by the co-inoculation of *Sinorhizobium meliloti*—Actinobacteria under salt stress. Curr. Microbiol..

[50] Mao J.-D., Johnson R.L., Lehmann J., Olk D.C., Neves E.G., Thompson M.L., Schmidt-Rohr K. (2012). Abundant and Stable Char Residues in Soils: Implications for Soil Fertility and Carbon Sequestration. Environ. Sci. Technol..

[51] Pinto O.H.B., Costa F.S., Rodrigues G.R., da Costa R.A., Fernandes G.D.R., Júnior O.R.P., Barreto C.C. (2021). Soil Acidobacteria Strain *AB23* Resistance to Oxidative Stress Through Production of Carotenoids. Microb. Ecol..

[52] Challacombe J.F., Hesse C.N., Bramer L.M., Ann M.L., Mary L., Samuel P., Carrie N., Verne G.G.L., Andrea P.A., Kuske C.R. (2019). Genomes and secretomes of Ascomycota fungi reveal diverse functions in plant biomass decomposition and pathogenesis. BMC Genom..

[53] Li M., Wei Y., Yin Y., Ding H., Zhu W., Zhou Y. (2022). The effect of intercropping mulberry (*Morus alba* L.) with peanut (*Arachis hypogaea* L.), on the soil rhizosphere microbial community. Forests.

[54] Shen G., Zhang S., Liu X., Jiang Q., Ding W. (2018). Soil acidification amendments change the rhizosphere bacterial community of tobacco in a bacterial wilt affected field. Appl. Microbiol. Biotechnol..

[55] Pal S., Ghosh S.K. (2014). Diversity of soil fungi in North 24 Parganas and their antagonistic potential against *Leucinodes orbonalis* Guen. (Shoot and fruit borer of brinjal). Environ. Monit. Assess..

[56] Ding T., Yan Z., Zhang W., Duan T. (2021). Green Manure Crops Affected Soil Chemical Properties and Fungal Diversity and Community of Apple Orchard in the Loess Plateau of China. J. Soil Sci. Plant Nutr..

[57] Zhang K., Cheng X., Shu X., Liu Y., Zhang Q. (2018). Linking soil bacterial and fungal communities to vegetation succession following agricultural abandonment. Plant Soil.

[58] Banakar S.P., Thippeswamy B., Thirumalesh B.V., Naveenkumar K.J. (2012). Diversity of soil fungi in dry deciduous forest of Bhadra Wildlife sanctuary, western Ghats of southern India. J. For. Res..

[59] Sanaei A., Sayer E.J., Yuan Z., Lin F., Fang S., Ye J., Liu S., Hao Z., Wang X. (2022). Soil Stoichiometry Mediates Links Between Tree Functional Diversity and Soil Microbial Diversity in a Temperate Forest. Ecosystems.

[60] Li H., Weng B.-S., Huang F.-Y., Su J.-Q., Yang X.-R. (2015). pH regulates ammonia-oxidizing bacteria and archaea in paddy soils in Southern China. Appl. Microbiol. Biotechnol..

[61] Sanyika T.W., Stafford W., Cowan D.A. (2012). The soil and plant determinants of community structures of the dominant actinobacteria in Marion Island terrestrial habitats, Sub-Antarctica. Polar Biol..

